# A249 PARADOXICAL PSORIASIS FOLLOWING ANTI-TNFΑ THERAPY IN INFLAMMATORY BOWEL DISEASE AND ASSOCIATION WITH AN IL-23 RECEPTOR VARIANT: A PRELIMINARY REPORT

**DOI:** 10.1093/jcag/gwad061.249

**Published:** 2024-02-14

**Authors:** N Natt, A Wilson

**Affiliations:** Gastroenterology, Western University, London, ON, Canada; Gastroenterology, Western University, London, ON, Canada

## Abstract

**Background:**

Patients with inflammatory bowel disease (IBD) who receive anti-tumor necrosis factor (TNF) α therapy are at risk of developing drug-related psoriatic skin lesions known as paradoxical psoriasis (PP). This is a severe unpredictable adverse drug event that often leads to discontinuation of anti-TNFα therapy. To date, there are no tools for identifying high-risk individuals. Variation in the IL-23 receptor (IL23R) gene has been linked to psoriasis onset and may be implicated in PP.

**Aims:**

To evaluate the frequency of the IL23R variant (*IL23R1142Gampersand:003EA*) in anti-TNFα exposed IBD patients who develop PP versus those who do not. Clinical variables associated with PP development will also be assessed.

**Methods:**

A case-control study is ongoing, including anti-TNFα-exposed adult IBD patients. Participants are divided based on the development of PP (cases n=11; controls n=48, identified to date, 500 additional patients will be screened). All participants were retrospectively genotyped for the occurrence of the *IL23R1142Gampersand:003EA* variant. Clinical variables and outcomes were assessed from the time of anti-TNFα exposure for at least one year of follow-up or to the time of anti-TNFα discontinuation.

**Results:**

To date, we have included 59 adult IBD patients treated with infliximab or adalimumab. Eleven patients developed PP after a median duration of treatment of three months with anti-TNFα therapy. The most common presentations were guttate, plaque, and pustular psoriasis. Six patients had PP that involved 50% or more of their body surface area (BSA). Nine participants discontinued their anti-TNFα therapy due to PP. None of the cases had a personal or family history of psoriasis. PP patients were similar to controls with respect to age, sex, ethnicity, IBD type, disease duration, and concurrent immunomodulator use. The variant genotype occurred more frequently in PP patients compared to controls (54.5% vs. 4.2%, OR 27.6, 95%CI 4.3-145.4). Those with the variant genotype also had more extensive psoriasis compared to wildtypes (mean BSA 61% vs. 25%, p=0.045).

**Conclusions:**

In this preliminary analysis, there was an increased frequency of the *IL23R1142Gampersand:003EA* variant in patients who developed PP compared to those who did not after anti-TNFα exposure. We did not identify any other clinical variables associated with development of PP. Interpretation of these results is limited by the small sample size. Completion of this study may help clarify the association between PP and the IL23R gene as well as other clinical risk factors implicated in PP development.

**Funding Agencies:**

None

